# A national, multicenter, secondary data use study evaluating efficacy and retention of first-line biologic treatment with tocilizumab in patients with rheumatoid arthritis in real-life setting: results from TURKBIO registry

**DOI:** 10.1038/s41598-022-26106-0

**Published:** 2022-12-20

**Authors:** Ayten Yazici, Özlem Özdemir Işık, Ediz Dalkılıç, Süleyman Serdar Koca, Yavuz Pehlivan, Soner Şenel, Nevsun Inanc, Servet Akar, Sema Yılmaz, Özgül Soysal Gündüz, Ayse Cefle, Ömer Fatih Karakaş, Fatos Onen

**Affiliations:** 1 Department of Internal Medicine Division of Rheumatology, Kocaeli University Faculty of Medicine, Kocaeli, Turkey; 2 Department of Internal Medicine Division of Rheumatology, Uludağ University Faculty of Medicine, Bursa, Turkey; 3 Department of Internal Medicine Division of Rheumatology, Fırat University Faculty of Medicine, Elazığ, Turkey; 4 Department of Internal Medicine Division of Rheumatology, Erciyes University Faculty of Medicine, Kayseri, Turkey; 5 Department of Internal Medicine Division of Rheumatology, Marmara University Faculty of Medicine, İstanbul, Turkey; 6 Department of Internal Medicine Division of Rheumatology, Katip Çelebi University Faculty of Medicine, İzmir, Turkey; 7 Department of Internal Medicine Division of Rheumatology, Selçuk University Faculty of Medicine, Konya, Turkey; 8 Department of Internal Medicine Division of Rheumatology, Celal Bayar University Faculty of Medicine, Manisa, Turkey; 9 Roche Turkey, İstanbul, Turkey; 10 Department of Internal Medicine Division of Rheumatology, Dokuz Eylül University Faculty of Medicine, İzmir, Turkey

**Keywords:** Rheumatology, Rheumatoid arthritis, Outcomes research

## Abstract

Tocilizumab (TCZ) is a recombinant humanized monoclonal antibody that targets the IL-6 receptor. TCZ found to be efficacious and has a good tolerated safety profile in rheumatoid arthritis (RA) patients. The aim of this study was to describe the disease activity and retention rate in Turkish RA patients who were prescribed TCZ as first-line biologic treatment in a real-world setting. Secondary data obtained from adult RA patients’ files was used in a multicenter and retrospective context. Clinical Disease Activity Index (CDAI), Disease Activity Score in 28 joints with ESR (DAS28-ESR), and retention rates of TCZ were evaluated at related time points. 130 patients (87.7% female) with a mean age of 53 years (SD; 15.0) were included in the study. Mean RA duration was 14 years and median duration of follow-up was 18.5 months. Number of patients with ongoing TCZ treatment at 6, 12, and 24 months were 121 (93%), 85 (65%), and 46 (35%), respectively. Remission rates at 6, 12, and 24 months per CDAI (< 2.8) and DAS28-ESR (< 2.6) scores were 61.5, 44.6, 30%, and 54.6, 40.8, 27.7%, respectively. Both CDAI and DAS28-ESR scores significantly improved at 6, 12 and 24 months (*p* < 0.001 for both). At 24 months, 23 patients (17.6%) discontinued TCZ, of whom majority (17/23) were due to unsatisfactory response. Retention rates of TCZ at 6, 12, and 24 months were 93, 84.3, and 72.2%, respectively. In this real-world study, TCZ as a first-line biologic therapy was found to be efficacious and showing high retention rates. These real-world study results are in line with previous randomized studies.

## Introduction

Rheumatoid arthritis (RA) is a chronic systemic inflammatory disease characterized by erosive synovitis that results in pain and swelling of peripheral joints, joint deformity, functional disability, and decreased health-related quality of life (QoL). In addition, the disease may involve extra-articular manifestations including skin, heart, lung, eye, nervous, renal, and gastrointestinal systems^1^. It is the most common inflammatory arthritis, affecting about 0.5–1% of the population in the United States and northern Europe^2^.

The goals of therapy for RA are to decrease joint inflammation and pain, preserve the ability of patients to function in activities of daily living and work, and prevent joint deformity and joint destruction. Optimal treatment regimen consists of a combined approach that includes both pharmacologic and non-pharmacologic therapies. Since complete recovery from RA is not possible, clinical remission is considered a good outcome^3^. Early treatment with conventional synthetic disease-modifying antirheumatic drugs (csDMARDs), such as methotrexate, has become the standard of care as several studies have suggested that it can slow down disease progression, and potentially induce clinical remission^4^. If the patient fails to respond adequately to methotrexate, the current standard approach is to add other synthetic or biological DMARD to the treatment regimen. Although the therapeutic efficacy and safety of tumour necrosis factor (TNF) inhibitors have been proven in a number of studies, it has been found that 30–40% of patients develop an inadequate response, either due to a lack of primary response or adverse events^5^. Moreover, most TNF inhibitors require concomitant methotrexate for maximum clinical efficacy, whereas tocilizumab (TCZ) has similar efficacy either it is combined with methotrexate or used as monotherapy^6^.

Tocilizumab (TCZ) is a recombinant humanized monoclonal antibody targeting the human IL-6 receptor. Intravenous TCZ (TCZ-IV) has been licensed in Europe in 2009, for the management of RA patients refractory to csDMARDs and TNF inhibitors, followed by the approval of subcutaneous TCZ (TCZ-SC) formulation in the year 2014^7^. TCZ-IV is approved in over 100 countries and has demonstrated efficacy with a well-established safety profile in patients with RA. Two phase III studies of TCZ, namely SATORI and SAMURAI studies, each have revealed the efficacy of TCZ monotherapy in RA patients who are refractory to treatment with DMARDs including methotrexate^8,9^. In a further randomized clinical trial, 69% of patients achieved an effective clinical response with a non-TNF biological vs. 52% of patients who took a second anti-TNF drug^10^.

The efficacy and safety of TCZ-SC has been evaluated in recent phase III clinical trials. SUMMACTA study evaluated the efficacy and safety of TCZ-SC in combination with DMARDs in patients with moderate-to-severe RA and inadequate response to one or more DMARD(s). The study demonstrated non-inferiority of TCZ-SC 162 mg weekly (qw) to TCZ-IV 8 mg/kg every 4 weeks (q4w) with regard to the American College of Rheumatology criteria (ACR20) response at week 24. TCZ-SC displayed a similar safety profile as TCZ-IV^11^.

This study was aimed to provide data regarding the retention rate, efficacy, and safety of TCZ use for RA in a real-world setting utilizing a patient registry.

## Methods

### Study design

This study was designed as a national, multicenter, retrospective, and non-interventional study in which data collection was conducted between March 2020 and June 2020 with contributions of 15 different rheumatology centers in Turkey. Patients with a diagnosis of RA who were receiving TCZ as first-line biological treatment with follow-up data of at least 6 months were included in the study. Secondary data were obtained from the TURKBIO Registry, which is a web-based registry of patients with rheumatic diseases receiving biologic DMARDs.

### Covariates

The primary objective was to assess the efficacy of TCZ as first-line biological treatment at 6, 12, and 24 months by mean change in Clinical Disease Activity Index (CDAI) and Disease Activity Score in 28 joints with ESR (DAS28-ESR) scores from baseline. Secondary objectives were to analyse the efficacy of TCZ by route of drug administration (TCZ-SC or TCZ-IV), to evaluate the retention rate and safety profile of TCZ over time, and to assess QoL by Health Assessment Questionnaire Disability Index (HAQ-DI) scores. Data were obtained from TURKBIO Registry for demographics, smoking status (current, previous or never), concomitant diseases, disease duration of RA, previous and concomitant csDMARD treatments, presence and number of swollen and tender joints, European Alliance of Associations for Rheumatology (EULAR) response rate (good or moderate), erythrocyte sedimentation rate (ESR), C-reactive protein (CRP) levels, CDAI score^12^, DAS28-ESR score^13^, HAQ-DI score^14^, date of TCZ treatment initiation and discontinuation, and adverse events. Disease activity per CDAI score was defined as remission (≤ 2.8), low disease activity (> 2.8 and ≤ 10), moderate disease activity (> 10 and ≤ 22) and high disease activity (> 22). Similarly, DAS28-ESR disease activity was defined as remission (< 2.6), low disease activity (≥ 2.6 and ≤ 3.2), moderate disease activity (> 3.2 and ≤ 5.1) and high disease activity (> 5.1).

### Statistical analysis

This was a secondary data use study of an existing registry, and therefore no study sample size was calculated. All data gathered during the course of the study were summarized using descriptive statistics; numeric variables were expressed as mean (standard deviation) or median (minimum and maximum or quartiles); categorical variables were expressed as number and percentage. Normality was assessed with visual (histogram and probability graphs) and analytical methods (Kolmogorov–Smirnov/Shapiro–Wilk tests). Kaplan–Meier estimates were calculated for the retention rate of TCZ. For comparison of 6, 12 and 24 month values of a continuous variable with the baseline data, the paired sample t test for matched pairs or, in case the normality assumption was questionable, the Wilcoxon signed rank test was used. Binary, multinomial and ordinal logistic regression analysis was carried out to evaluate the effects of demographic and baseline disease characteristics on the retention of TCZ treatment. Drug retention was selected as the dependent variable, and age, gender, concomitant diseases, smoking status, concomitant csDMARD treatments, baseline disease activity scores and baseline joint status were selected as independent variables. For statistical analysis, PASW 18.0 for Windows was used. A *p* value of < 0.05 was considered significant.

### Ethics approval and consent to participate

The TURKBIO registry setup was approved by the Ministry of Health in July 2013 and this study was approved by the Non-Interventional Clinical Research Ethics Committee of Kocaeli University on May 14, 2020, with an approval number: KİA 2020/100. Participants gave written informed consent for TURKBIO registry, and an additional consent was exempted due to the retrospective nature of this study. The study was performed in accordance with the Declaration of Helsinki and relevant guidelines/regulations.

## Results

The study included 130 RA patients (87.7% female) with a mean age of 53 (15) years. The number of patients with ongoing tocilizumab treatment and follow-up at 6, 12, and 24 months were 121 (93%), 85 (65%) and 46 (35%), respectively. Median duration of follow-up was 18.55 (4.04–78.55) months. Majority (90.8%) of patients were given tocilizumab via intravenous route at baseline. At TCZ initiation, one (0.8%) patient had remission per CDAI score while no patient was in remission per DAS28-ESR score. Patient demographics and baseline disease and treatment characteristics are presented in Table 1.

**Table 1:** Patient demographics and baseline disease and treatment characteristics.

**Characteristics**	
Age (years), mean (SD)	53 (15)
Female, n (%)	114 (87.7)
Duration of RA (years), mean (SD)^a^	14.0 (8.0)
BMI (kg/m^2^), mean (SD)^b^	28.3 (5.1)
**Smoking status, n (%)**	15 (11.5)
Never smoked	86 (66.2)
Current smoker	15 (11.5)
**Concomitant Diseases, n (%)**	
Hypertension	21 (16.2)
Osteoporosis	11 (8.5)
Diabetes mellitus	8 (6.2)
Asthma	5 (3.8)
Depression	5 (3.8)
Coronary artery disease	2 (1.5)
Hypercholesterolemia	2 (1.5)
Other cardiovascular diseases	2 (1.5)
Inflammatory bowel disease	2 (1.5)
Renal disease	2 (1.5)
ESR (mm/hr), mean (SD)^c^	45.0 (26.0)
CRP (mg/L), mean (SD)^d^	32.6 (39.6)
**Disease activity per CDAI score, n (%)**^**e**^	
Remission	1 (0.8)
Low disease activity	14 (11.1)
Moderate disease activity	64 (50.8)
High disease activity	47 (37.3)
**Disease activity per DAS28-ESR score, n (%)**^**f**^	
Remission	0 (0)
Low disease activity	0 (0)
Moderate disease activity	60 (47.6)
High disease activity	66 (52.4)
**Route of TCZ administration, n (%)**	
Intravenous	118 (90.8)
Subcutaneous	12 (9.2)
**Concurrent csDMARD treatments, n(%)**	
Methotrexate	33 (25.4)
Leflunomide	33 (25.4)
Chloroquine	19 (14.6)
Sulphasalazine	10 (7.7)

^a^For duration of years, n = 127.

^b^For BMI, n = 73.

^c^For ESR, n = 126.

^d^For CRP, n = 127.

^e^For disease activity per CDAI score, n = 126.

^f^For disease activity per DAS28-ESR score, n = 126.

*BMI* Body Mass Index, *CDAI* Clinical Disease Activity Index, *CRP* C-Reactive Protein, *csDMARD* Conventional Synthetic Disease-Modifying Antirheumatic Drug, *DAS28-ESR* Disease Activity Score in 28 joints-Erythrocyte Sedimentation Rate, *ESR* Erythrocyte Sedimentation Rate, *RA* Rheumatoid Arthritis, *SD* Standard Deviation, *TCZ* Tocilizumab.

CDAI, DAS28-ESR, and HAQ-DI scores were significantly improved at 6, 12 and 24 months compared to baseline (*p* < 0.001 for all, Table 2). Remission rates at 6, 12, and 24 months per CDAI and DAS28-ESR scores were 61.5, 44.6, 30%, and 54.6, 40.8, 27.7%, respectively. Similarly, 74.8%, 82.5% and 86.4% of patients achieved EULAR good response while 13.9, 11.3 and 9.1% additionally achieved EULAR moderate response at 6 months (n = 115), 12 months (n = 80), and 24 months (n = 44), respectively.

**Table 2:** Pairwise disease activity and quality of life scores at baseline, 6, 12, and 24 months.

Survey	Pair	N	Median (Min–Max)	*p* value*
CDAI	Baseline	113	19.5 (0–47.0)	< 0.001
	6 months		2.0 (1.0–24.5)	
	Baseline	74	20.1 (8.4–47.0)	< 0.001
	12 months		1.8 (1.0–5.2)	
	Baseline	41	17.2 (9.0–38.0)	< 0.001
	24 months		1.7 (1.2–3.7)	
DAS28-ESR	Baseline	112	5.2 (3.3–7.2)	< 0.001
	6 months		2.2 (0.3–6.7)	
	Baseline	78	5.3 (3.4–7.2)	< 0.001
	12 months		2.0 (0–5.2)	
	Baseline	41	5.2 (3.4–6.9)	< 0.001
	24 months		1.8 (0–3.6)	
HAQ-DI	Baseline	118	0.95 (0–2.875)	< 0.001
	6 months		0.5 (0–2.0)	
	Baseline	81	1.0 (0–3.0)	< 0.001
	12 months		0.25 (0–2.5)	
	Baseline	43	0.875 (0–2.125)	< 0.001
	24 months		0 (0–1.13)	

*Wilcoxon Signed Rank Test.

*CDAI* Clinical Disease Activity Index, *DAS28-ESR* Disease Activity Score in 28 joints-Erythrocyte Sedimentation Rate, *HAQ-DI* Health Assessment Questionnaire-Disability Index.

Of 118 patients who were using TCZ-IV, the decrease in CDAI and DAS28-ESR scores at 6, 12 and 24 months was statistically significant (*p* < 0.001 for all). In patients using TCZ-SC, only the decrease in CDAI and DAS28-ESR scores at 6 months was statistically significant in comparison to baseline scores (*p* = 0.003 for both). The change in disease activity scores per route of tocilizumab administration is given in Table 3. During the study, the number of patients who switched from TCZ-IV to TCZ-SC was four. None of the patients switched from TCZ-SC to TCZ-IV.

**Table 3:** The change in disease activity scores per route of tocilizumab administration.

Survey	Pair	TCZ-IV			TCZ-SC		
		N	Median (Min–Max)	P*	N	Median (Min–Max)	P*
CDAI	Baseline	102	19.9 (0–47.0)	< 0.001	11	14.0 (3.2–36.0)	0.003
	6 months		2.0 (1.0–24.5)			1.8 (1.1–3.2)	
	Baseline	72	20.0 (8.4–47.0)	< 0.001	2	27.3 (24.0–30.6)	–**
	12 months		1.8 (1.0–5.2)			2.8 (1.5–4.0)	
	Baseline	41	17.2 (9.0–38.0)	< 0.001	0	–	–**
	24 months		1.7 (1.2–3.7)			–	
DAS28-ESR	Baseline	101	5.2 (3.3–7.2)	< 0.001	11	4.3 (3.5–6.3)	0.003
	6 months		2.3 (0.3–6.7)			1.3 (0.8–3.6)	
	Baseline	76	5.2 (3.4–7.2)	< 0.001	2	6.0 (5.7–6.3)	–**
	12 months		2.0 (0–5.2)			2.5 (1.0–4.0)	
	Baseline	41	5.2 (3.4–6.9)	< 0.001	0	–	–**
	24 months		1.8 (0–3.6)			–	

*Wilcoxon Signed Rank Test.

**Analysis could not be done as patient number is inadequate.

*TCZ-IV* Intravenous route of tocilizumab administration, *TCZ-SC* Subcutaneous route of tocilizumab administration, *CDAI* Clinical Disease Activity Index, *DAS28-ESR* Disease Activity Score in 28 joints-Erythrocyte Sedimentation Rate.

**Figure 1:**
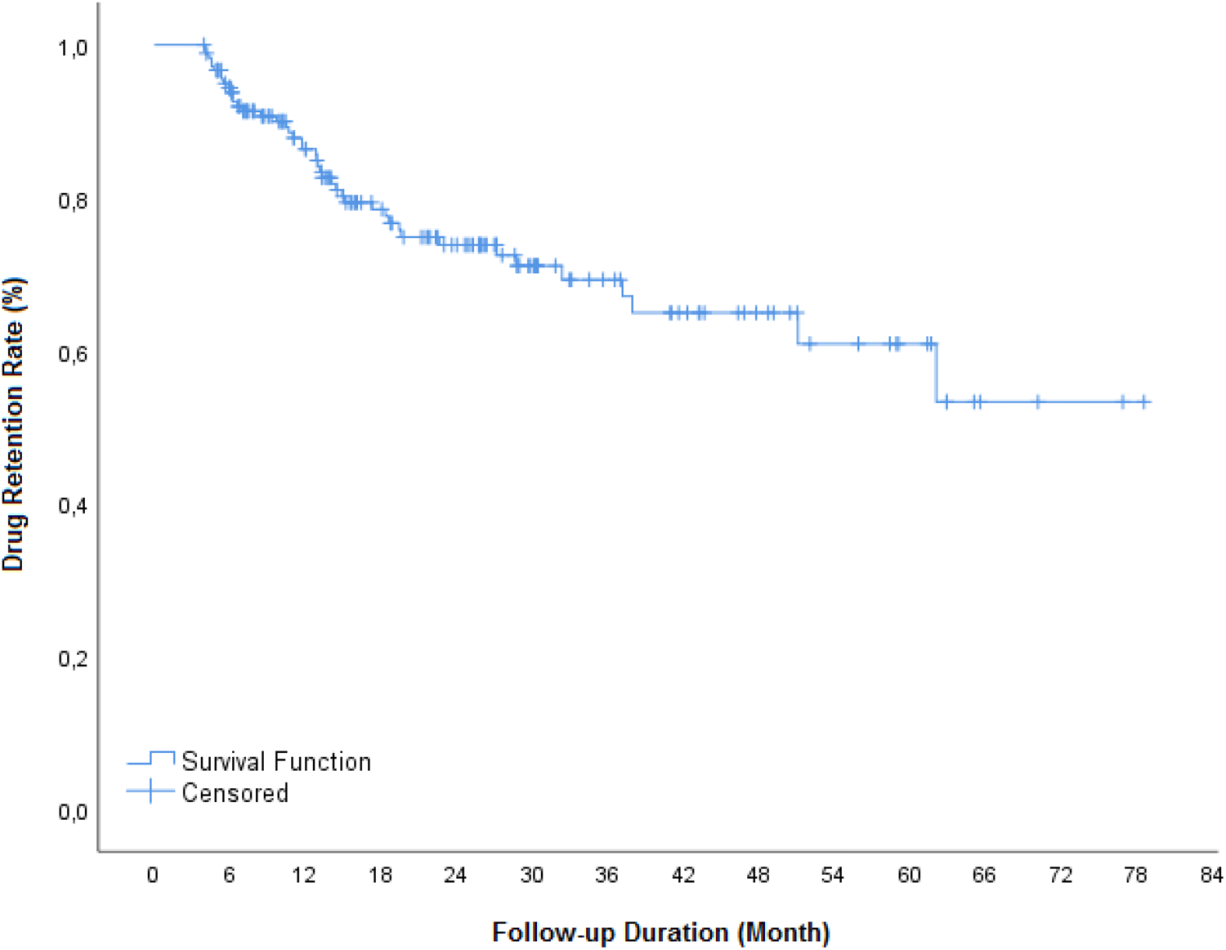
Tocilizumab retention rates.

TCZ retention rates at 6, 12 and 24 months were 93, 84.3 and 72.2%, respectively (Fig. 1). Regression analysis showed that absence of concomitant methotrexate or leflunomide treatments independently associated with better assumption of TCZ retention [β = −0.92, 95%CI (0.17–0.92), *p* = 0.033, for both]. At 24 months, 23 patients (17.6%) discontinued their medications, 17 of them due to unsatisfactory response. Overall, 13 adverse events were reported (infection in two, rash in two, allergy in two, tuberculosis in one, and other adverse events in six patients).

## Discussion

In this study, the data from TURKBIO Registry were gathered with the aim to evaluate TCZ use as first-line biological treatment in patients with RA in a real-world setting. Our results indicated response rates similar to the ones found in TCZ phase III trials with high retention rates and a good safety profile^8,9^. Our patient population revealed a moderate to severe RA at baseline for almost all patients per DAS28-ESR scores and almost 90% of the patients had moderate to high disease activity per CDAI scores. Overall, there was a significant decrease in CDAI and DAS28-ESR scores at 6, 12 and 24 months compared to baseline. At six months after the initiation of TCZ treatment nearly 75% of the patients had EULAR good response. Regarding the real-life studies, the proportion of first line TCZ users who achieved DAS28-ESR remission was 51 and 52% at 6th and 24th months, respectively in the Italian biologics’ registry Gruppo Italiano Studio Early Arthritis (GISEA)^15^. The rate of EULAR good responders in the 6th month was found 42% in the British Society for Rheumatology Biologics Registry for RA (BSRBR-RA)^16^. In terms of 6th month DAS28-ESR results, our study was found to be similar with other real-life studies. However, although the median DAS28-ESR scores decreased in time, the proportion of patients who achieved remission at one and two years was lower than the proportion of patients who achieved remission at 6 months, which is not consistent with many real-life data. The reason for this difference may be the decrease in the number of patients followed up at the 12th and 24th months in our study.

The decreases in HAQ-DI scores at 6, 12 and 24 months of TCZ treatment were also found to be significant in comparison to baseline scores. These findings are in line with the effectiveness and QoL results obtained from several large randomized clinical trials, which established the efficacy of TCZ-IV monotherapy or combination therapy with csDMARDs^17^.

The majority of patients were given TCZ treatment via an intravenous route at baseline (90.8%), which strongly indicates that TCZ-IV is the more commonly preferred formulation in routine clinical practice in Turkey. The reason behind this may be that either TCZ-IV has demonstrated efficacy with a well-established safety profile in a range of clinical trials or TCZ-SC is approved for RA patients several years after TCZ-IV^18^. Despite the low number of patients administered with TCZ-SC, we found similar disease activity at 6 and 12 months in TCZ-IV and TCZ-SC subgroups. Only four patients switched from IV to SC formulation which also may indicate a preference for IV administration of TCZ.

In the current study, at two years almost 75% of the patients were still on TCZ treatment, which were considered as satisfactory for drug retention. The drug continuation at one year was estimated lower (64%) in the Swedish registry^19^, however, the rate (77%) was found similar in the BSRBR-RA registry^16^. In a recent study, in which data from 16 countries were collected to evaluate efficacy, safety and usage patterns of TCZ in real life settings, Kaplan–Meier estimates for the proportions of patients still receiving TCZ at 24 weeks were 81.3% for biologic-naive patients, which was also lower than our study^20^. However, given the low number of followed-up patients in our study, these results should be approached with caution.

Despite recorded discontinuation of the concomitant medications, namely methotrexate and leflunomide, patients still continued with TCZ treatment. Even though methotrexate is indispensable in the treatment of RA, TCZ monotherapy seems to occupy a significant place in the management of RA patients who had an inadequate response or who develop intolerance to methotrexate and other DMARDs^21^. The safety profile of TCZ based on adverse events was also evaluated, and overall, no unexpected adverse events were seen. TCZ was considered to be a tolerable biological DMARD.

Our study has a few limitations. The number of patients using TCZ-SC was limited. Therefore, it is not possible to implicate definite results. Since a retrospective patient registry was used as a data source in the study, data from all patients were not available for several variables.

## Conclusion

The results of the present study showed that TCZ, either IV or SC formulation, as first-line biologic treatment is an effective and safe treatment option with a good quality of life in RA patients.

## Data Availability

All data generated or analysed during this study are included in this published article.

## Abbreviations

CDAI: Clinical Disease Activity Index
CRP: C-reactive protein
csDMARDs: Conventional synthetic disease-modifying anti-rheumatic drugs
DAS28-ESR: Disease activity score in 28 joints-Erythrocyte Sedimentation Rate
DMARDs: Disease-modifying anti-rheumatic drugs
ESR: Erythrocyte sedimentation rate
EULAR: European Alliance of Associations for Rheumatology
HAQ-DI: Health Assessment Questionnaire-Disability Index
IV: Intravenous
QoL: Quality of life
RA: Rheumatoid arthritis
SC: Subcutaneous
TCZ: Tocilizumab
TNF: Tumor necrosis factor
